# COVID-19 Inpatient Deaths and Brought-in-Dead Cases in Malaysia

**DOI:** 10.3389/fpubh.2022.872838

**Published:** 2022-07-06

**Authors:** Poh Ying Lim, Salmiah Md Said, Hayati Kadir Shahar, Ahmad Zaid Fattah Azman, Siti Aisah Mokhtar, Aidalina Mahmud

**Affiliations:** ^1^Department of Community Health, Faculty of Medicine and Health Sciences, Universiti Putra Malaysia, Serdang, Malaysia; ^2^Malaysian Research Institute of Ageing (MyAgeing), Universiti Putra Malaysia, Serdang, Malaysia

**Keywords:** COVID-19, mortality case, brought-in-death, inpatient death, Malaysia

## Abstract

Coronavirus disease 2019 (COVID-19) deaths can occur in hospitals or otherwise. In Malaysia, COVID-19 deaths occurring outside of the hospital and subsequently brought to the hospital are known as brought-in-dead (BID) cases. To date, the characteristics of BID COVID-19 cases in Malaysia are not clear. The objectives of this study are 2-fold: to explore the characteristics of 29,155 mortality cases in Malaysia and determine the factors associated with the high probability of BID, using the multilevel logistic regression model. Data on COVID-19 mortality cases from the entire country between March 17, 2020 and November 3, 2021 were retrieved from a national open data source. Of the 29,155 COVID-19 mortality cases, 5,903 (20.2%) were BID. A higher probability of BID (*p* < 0.05) was seen among individuals aged between 18 and 59 years, non-Malaysians, had no comorbidities, did not receive COVID-19 vaccination, and the interval between the date of death and diagnosis. A high prevalence of BID is an alarming public health issue, as this may signal health system failure at one or several levels and, hence, need urgent attention from relevant stakeholders. Based on the findings of this study, increasing the intensity of the vaccination campaign, addressing any issues faced by noncitizens about to COVID-19 management in- and out-of-hospital, increasing the awareness of signs and symptoms of worsening COVID-19 and, hence, the significance of self-monitoring, and determining the potential gaps in the health system may contribute to their increased risk of deaths.

## Introduction

As of December 2021, Coronavirus disease 2019 (COVID-19) mortality cases in the world reached approximately 5.33 million. Many factors contribute to the survival, and, hence, death rates, of patients with COVID-19. Among them are inadequate human resources and inadequate medical supplies such as medicines, equipment, and facilities such as intensive care beds (1–3).

In Malaysia, the first COVID-19 death was reported on 17 March 2020. Since then, the country has recorded fluctuating numbers of COVID-19 deaths and by December 2021, there were approximately 30,000 people died of COVID-19 in the country. From the beginning of the COVID-19 pandemic, several measures have been implemented by the Malaysian government in reducing the spread of COVID-19 and its related morbidity and mortality. Among these measures was the Movement Control Order (MCO), which ranged from total movement restrictions (lockdown) to partial restrictions (such as allowing crucial sectors and businesses to operate) (4). In addition to controlling the movement of the population, the government consistently urged the public, especially the vulnerable groups (children, elderly, unvaccinated individuals, and people with comorbidities), to stay away from crowded places and to strictly adhere to the national COVID-19 standard operating procedure (SOP) because high infection and mortality rates have been reported among these vulnerable groups (2, 5–9).

In general, COVID-19 mortality cases can be classified into two categories based on the location of death: within the hospital or out-of-hospital (5). Studies show that the percentage of COVID-19 cases, which occurred out-of-hospital, namely, at home, in several developed nations (England, Wales, Scotland, Northern Ireland, and the United States of America) ranged between 4.0% and 8.1% (1, 6, 7). A study in Zambia, however, showed that the out-of-hospital deaths were almost 10 times higher at 72.5% in August 2020 (10).

In Malaysia, COVID-19 deaths occurring out-of-hospital are referred to as brought-in-dead (BID) cases and must fulfill several criteria set by the Ministry of Health (MOH) Malaysia (5). The criteria include confirmation of COVID-19 diagnosis through laboratory tests such as rapid test (RT) and PCR, the presence of an epidemiological link between the deceased and other COVID-19 cases, or the presence of radiological changes suggestive of COVID-19 infection, if an autopsy was not conducted (5). To date, the sociodemographic and clinical characteristics of COVID-19 deaths, particularly the BID COVID-19 cases, remain unclear.

As knowledge of the sociodemographic and clinical characteristics of patients who died due to COVID-19 is needed for a better understanding and proper management of the disease, this study aims to explore these characteristics and determine the factors associated with the high probability of BID COVID-19 cases in Malaysia.

### Data Source and Methodology

This study was a retrospective record review. All the patients confirmed to have died due to COVID-19 in Malaysia, between March 17, 2020 and November 3, 2021, were included in this study. Data retrieved from the national open data database were on the date of death date of confirmed COVID-19 diagnosis (from which the interval between the date of death and confirmation of COVID-19 was determined), vaccination status (no vaccination, 1 dose and 2 doses of vaccination), date of vaccination(s), type of vaccine, comorbidity status (yes or no), nationality (Malaysian or non-Malaysian), age, gender (female or male), place of death (inpatient death or BID), and state [Johor, Kedah, Kelantan, Melaka, Negeri Sembilan, Pahang, Perak, Perlis, Pulau Pinang, Sabah, Sarawak, Terengganu, Wilayah Persekutuan Kuala Lumpur (WP Kuala Lumpur), Wilayah Persekutuan Labuan (WP Labuan), and Wilayah Persekutuan Putrajaya (WP Putrajaya)] (https://github.com/MoH-Malaysia/covid19-public/tree/main/epidemic).

Descriptive analysis was conducted to determine the profile of all the COVID-19 mortality cases. The profiles of inpatient mortality cases and the BID cases were also compared. The trend of COVID-19 mortality cases was demonstrated based on the epidemiological weeks (also known as the Epid Week). A multiple multilevel logistic regression analysis was conducted to investigate the factors associated with places of death: inpatient death was coded as zero and BID cases were coded as one. A multiple multilevel logistic regression model with two levels, individual mortality cases nested within each state, was used as follows:

$$\begin{array}{r} y_{ij}=\alpha_{ij}+\beta_{ij}X_{ij}+\mu_{j}\\ \mu_{j}\sim N\left(0,\;\sigma^{2}\right) \end{array}$$

where *y*_*ij*_ was the binary outcome with zero (inpatient death) or one (BID), α_*ij*_ was an intercept of the equation, and *i* and *j* represented the *ith* mortality cases of the *jth* state, respectively. β_*ij*_ was the coefficient of variables *X*_*ij*_ and μ_*j*_ was the random effect of state level, which the variation σ^2^. The model was set up using MLwiN version 2.25 (the Centre for Multilevel Modelling, University of Bristol), estimated using quasi-likelihood methods. Variables with *p* < 0.05 were retained in the final model. Confounder, multicollinearity between variables, and interaction terms between variables were also checked.

## Results

As of November 3, 2021, there were 29,155 COVID-19 mortality cases in Malaysia (Table 1). The majority of the cases were from Selangor (*n* = 9,620, 33.0%), the most populous state in the country, followed by Johore (3,679, 12.6%) and WP Kuala Lumpur (2,563, 8.8%), both of which are also major cities, located in the West Coast and Southern Region of Peninsular Malaysia, respectively. The majority of the deceased were not vaccinated (19,698, 67.6%), had one or more comorbidity (22,376, 76.7%), and were aged 60 years and more (16,230, 55.7%).

**Table 1:** Distribution of the COVID-19 mortality cases (*N* = 29,155).

**Variables**		**Frequency (*n*)**	**Percentage (%)**
Year	2020	514	1.8
	2021	28641	98.2
State	Johor	3679	12.6
	Kedah	1995	6.8
	Kelantan	1054	3.6
	Melaka	905	3.1
	Negeri Sembilan	1239	4.2
	Pahang	664	2.3
	Pulau Pinang	1611	5.5
	Perak	1130	3.9
	Perlis	118	0.4
	Selangor	9620	33.0
	Terengganu	468	1.6
	Sabah	2540	8.7
	Sarawak	1399	4.8
	W.P. Kuala Lumpur	2563	8.8
	W.P. Labuan	149	0.5
	W.P. Putrajaya	21	0.1
Age	0–11	70	0.2
	12–17	45	0.2
	18–59	12810	43.9
	60+	16230	55.7
Gender	Female	12391	42.5
	Male	16764	57.5
Malaysian	Non–Malaysian	3703	12.7
	Malaysian	25452	87.3
Comorbidity status	No comorbidity	6779	23.3
	Comorbidity	22376	76.7
Vaccination status	No vaccination	19698	67.6
	1 dose	5339	18.3
	2 doses	4118	14.1
Type of vaccination received (*n* = 9457)	Pfizer	2991	31.6
	Sinovac	5692	60.2
	AstraZeneca	767	8.1
	Others	7	0.1
Place of death	Inpatient death	23252	79.8
	BID	5903	20.2
Interval after confirmed to death (days)	0	8710	29.9
	1–3	4481	15.4
	4–7	5558	19.1
	8–14	6560	22.5
	15–21	2602	8.9
	22–28	764	2.6
	29–35	245	0.8
	36–42	93	0.3
	43–49	57	0.2
	50 above	85	0.3

Of the total mortality cases, 20.2% of them were BID cases (5,903). The majority of cases with the interval between the date of death and the confirmation of COVID-19 status ranged between 1 and 14 days (16,599, 57.0%). Of these, 15.4% (4,481) of cases were confirmed as COVID-19 positive between 1 and 3 days, 19.1% (5,558) of cases were confirmed as COVID-19 positive between 4 and 7 days, and 22.5% (6,560) of cases were confirmed as COVID-19 positive between 8 and 14 days. There were 8,710 cases (29.9%) that were confirmed COVID-19 positive on the same date of death. These cases were tested for COVID-19 upon arrival at the hospital, if the status was not already known.

**Figure 1:**
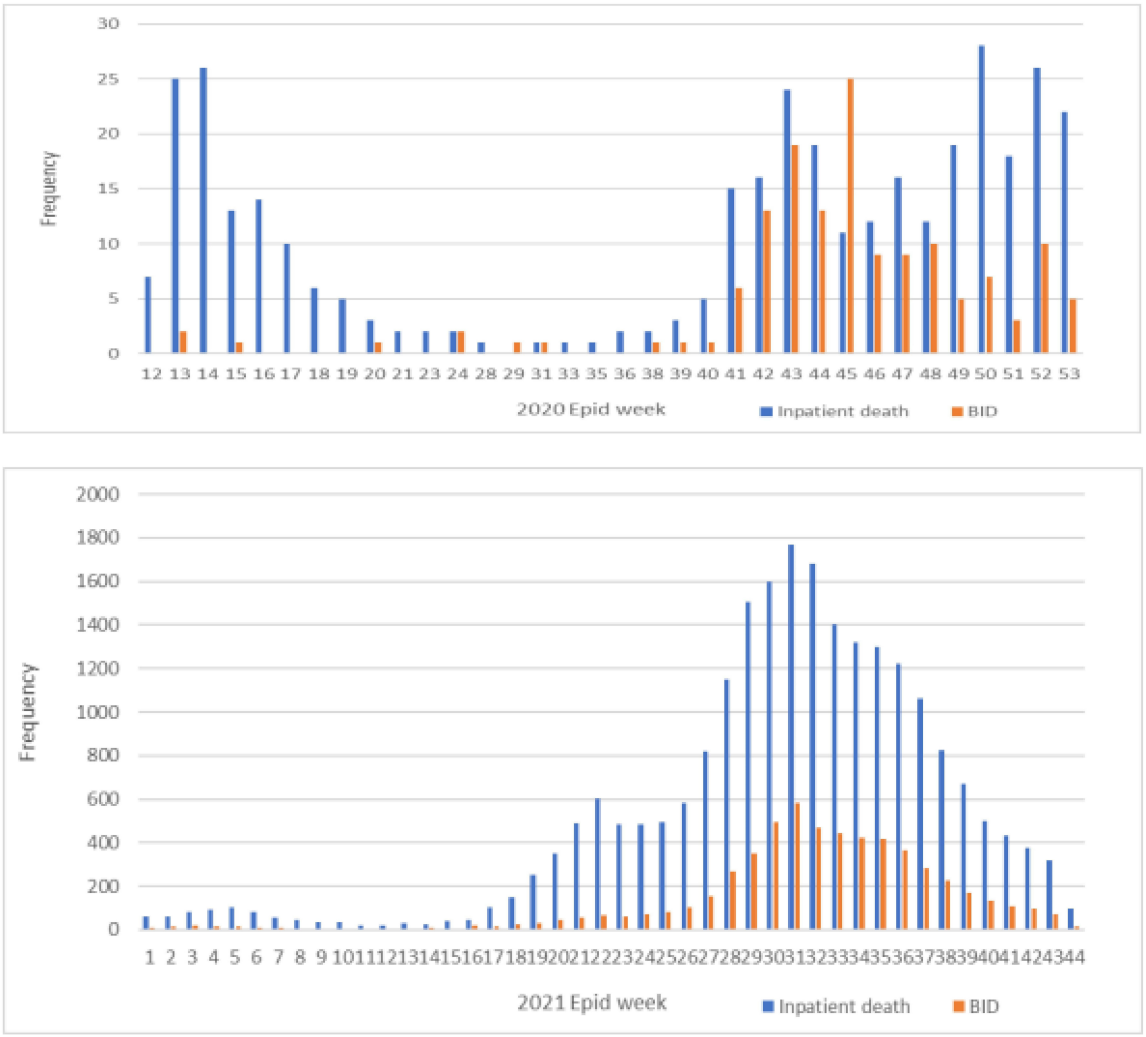
Distribution of inpatient death and the brought-in-dead (BID) COVID-19 mortality cases between the years 2020 and 2021.

The distribution of inpatient deaths and the BID COVID-19 deaths in the years 2020 and 2021 is shown in Figure 1. The majority of the cases were in the year 2021, which peaked between the 30th and 32nd Epid Week (25 July−14 August 2021).

As with the data on the overall COVID-19 death, the majority of BID cases were also noted in Selangor (34.7%) and Kuala Lumpur (11.3%). However, a large number of BID cases was also detected in Sabah (16.7%), which is a state located on the island of Borneo and is one of the less developed and has lower socioeconomic status compared to the other states in the country.

The adult BID cases were of higher proportion among those in the younger age group, where more than half (54.5%) were aged 18–59 years, compared to those in the same age group who died in hospital (41.3%). The inpatient COVID-19 deaths were more common among patients aged 60 years or more, who constituted almost 60% of these deaths. Similarly, in the pediatric age group (0–11 years), the proportion of those who were BID was higher than those who died at the hospital (0.4 vs 0.2%).

The data also showed that more than 60% of BID cases had comorbidities compared to a higher proportion of inpatient death cases with comorbidities (80.8%). Approximately one-third (31.9%) of the BID cases were non-Malaysians. The majority of the BID cases (79.3%) were confirmed as COVID-19 at the same date of death (Table 2).

**Table 2:** Comparison characteristics of mortality cases between inpatient death and brought–in–dead (BID).

**Variable**		**Place of death**				**Total**	**χ^2^ value**	**P-value**
		**Inpatient death**		**BID**				
		**n**	**%**	**n**	**%**			
Year	2020	369	1.6	145	2.5	514	20.547	<0.001^*^
	2021	22883	98.4	5758	97.5	28641		
State	Johor	3213	13.8	466	7.9	3679	975.180	<0.001^*^
	Kedah	1689	7.3	306	5.2	1995		
	Kelantan	827	3.6	227	3.8	1054		
	Melaka	789	3.4	116	2.0	905		
	Negeri Sembilan	1107	4.8	132	2.2	1239		
	Pahang	581	2.5	83	1.4	664		
	Pulau Pinang	1260	5.4	351	5.9	1611		
	Perak	981	4.2	149	2.5	1130		
	Perlis	112	0.5	6	0.1	118		
	Selangor	7570	32.6	2050	34.7	9620		
	Terengganu	419	1.8	49	0.8	468		
	Sabah	1557	6.7	983	16.7	2540		
	Sarawak	1125	4.8	274	4.6	1399		
	W.P. Kuala Lumpur	1895	8.1	668	11.3	2563		
	W.P. Labuan	107	0.5	42	0.7	149		
	W.P. Putrajaya	20	0.1	1	0.0	21		
Age	0–11	44	0.2	26	0.4	70	354.561	<0.001^*^
	12–17	33	0.1	12	0.2	45		
	18–59	9595	41.3	3215	54.5	12810		
	60+	13580	58.4	2650	44.9	16230		
Gender	Female	9904	42.6	2487	42.1	12391	0.413	0.520
	Male	13348	57.4	3416	57.9	16764		
Malaysian	Non-Malaysian	1820	7.8	1883	31.9	3703	2460.288	<0.001^*^
	Malaysian	21432	92.2	4020	68.1	25452		
Comorbidity status	No comorbidity	4463	19.2	2316	39.2	6779	1059.51	<0.001^*^
	Comorbidity	18789	80.8	3587	60.8	22376		
Vaccination status	No vaccination	15414	66.3	4284	72.6	19698	10.488	<0.001^*^
	1 dose	4511	19.4	828	14.0	5339		
	2 doses	3327	14.3	791	13.4	4118		
Type of vaccination received (*n* = 9457)	Pfizer	2488	31.7	503	31.1	2991	17.349	0.001^*^
	Sinovac	4669	59.6	1023	63.2	5692		
	AstraZeneca	675	8.6	92	5.7	767		
	Others	6	0.1	1	0.1	7		
Interval after confirmed to death (days)	0	4029	17.3	4681	79.3	8710	8762.688	<0.001^*^
	1–3	4007	17.2	474	8.0	4481		
	4–7	5237	22.5	321	5.4	5558		
	8–14	6308	27.1	252	4.3	6560		
	15–21	2530	10.9	72	1.2	2602		
	22–28	733	3.2	31	0.5	764		
	29–35	215	0.9	30	0.5	245		
	36–42	86	0.4	7	0.1	93		
	43–49	43	0.2	14	0.2	57		
	50 above	64	0.3	21	0.4	85		

* *P < 0.05; χ^2^ value, chi square test value*.

The univariate multilevel logistic regression model was used to investigate the association of each independent variable associated with the probability of BID. The results showed that associations between all the independent variables and BID status were statistically significant, except for gender (Table 3). All the variables were tested in multiple multilevel logistic regression model to identify the factors associated with the probability to BID, using the stepwise variable selection method. Variable with *p* > 0.05 was not retained in the multiple multilevel logistic regression model.

**Table 3:** Factors associated with probability to BID using the univariate multilevel logistic regression model.

**Variable**	**Unadjusted coefficient**	**SE**	**Crude OR**	**95% CI of OR**		**P–value**
**Vaccination status**						
No vaccination	Ref.					
1 dose	−0.480	0.045	0.619	0.567	0.676	<0.001^*^
2 doses	−0.201	0.047	0.818	0.746	0.897	<0.001^*^
**Age**						
18–59	0.531	0.031	1.701	1.600	1.807	<0.001^*^
0–11	0.954	0.263	2.596	1.550	4.347	<0.001^*^
12–17	0.582	0.353	1.790	0.896	3.575	0.099
60+	Ref.					
**Gender**						
Female	Ref.					
Male	0.001	0.031	1.001	0.942	1.064	0.974
**Malaysian**						
No	1.622	0.039	5.063	4.691	5.465	<0.001^*^
Yes	Ref.					
**Comorbidity status**						
No comorbidity	0.955	0.033	2.599	2.436	2.772	<0.001^*^
Comorbidity	Ref.					
**Interval after confirmed to death (days)** ^a^						
1–3	Ref.					
4–7	−0.644	0.078	0.525	0.451	0.612	<0.001^*^
8–14	−1.074	0.083	0.342	0.290	0.402	<0.001^*^
15–21	−1.420	0.133	0.242	0.186	0.314	<0.001^*^
22–28	−1.042	0.198	0.353	0.239	0.520	<0.001^*^
29–35	0.107	0.213	1.113	0.733	1.690	0.615
36–42	−0.363	0.408	0.696	0.313	1.548	0.374
43–49	1.084	0.318	2.956	1.585	5.514	0.001^*^
50 above	1.053	0.264	2.866	1.708	4.809	<0.001^*^

* *P < 0.05, SE, Standard Error; CI, Confidence Interval; OR, Odd Ratios; Ref, Reference group*.

a *There was 8710 cases (29.9%) had the same date of confirmed positive and date of death, which might be these cases were tested COVID−19 upon arrival at the hospital. Therefore, interval between actual confirmed positive with death date remain unknown and were not included in the analysis*.

The results show that higher probabilities of BID were related to the young age groups (0–11 and 18–59 years), being non-Malaysian, and having no known comorbidities, whereas lower probabilities of BID were related to having had at least one dose or two doses of COVID-19 vaccine and having the longer interval between death and confirmation of COVID-19 diagnosis (Table 4).

**Table 4:** Factors associated with probability to BID using the multiple multilevel logistic regression model.

**Variable**	**Adjusted coefficient**	**SE**	**Adjusted OR**	**95% CI of OR**		**P–value**
Intercept	−2.540	0.058				
**Malaysian**						
No	0.939	0.051	2.557	2.314	2.826	<0.001^*^
Yes	Ref.					
**Vaccination status**						
No vaccination	Ref.					
1 dose	−0.203	0.052	0.816	0.737	0.904	<0.001^*^
2 doses	0.076	0.055	1.079	0.969	1.202	0.167
**Age**						
18–59	0.198	0.040	1.219	1.127	1.318	<0.001^*^
0–11	0.639	0.327	1.895	0.998	3.596	0.051
12–17	0.494	0.428	1.639	0.708	3.792	0.248
60+	Ref.					
**Comorbidity status**						
No comorbidity	0.707	0.042	2.028	1.868	2.202	<0.001^*^
Comorbidity	Ref.					
**Interval after confirmed to death (days)** ^a^						
1–3	Ref.					
4–7	−0.635	0.078	0.530	0.455	0.617	<0.001^*^
8–14	−1.069	0.083	0.343	0.292	0.404	<0.001^*^
15–21	−1.423	0.132	0.241	0.186	0.312	<0.001^*^
22–28	−1.05	0.194	0.350	0.239	0.512	<0.001^*^
29–35	0.098	0.213	1.103	0.727	1.674	0.645
36–42	−0.364	0.4	0.695	0.317	1.522	0.363
43–49	1.125	0.321	3.080	1.642	5.779	<0.001^*^
50 above	0.993	0.269	2.699	1.593	4.573	<0.001^*^

* *P < 0.05; SE, Standard Error; CI, Confidence Interval; OR, Odd Ratios; Ref, Reference group. Random effect for state level: 0.279 (0.031), stepwise variable selection method*.

a *There was 8710 cases (29.9%) had the same date of confirmed positive and date of death, which might be these cases were tested COVID−19 upon arrival at the hospital. Therefore, interval between actual confirmed positive with death date remain unknown and were not included in the analysis*.

## Discussion

### Characteristics of COVID-19 Deaths in Malaysia

In Malaysia, the prevalence of COVID-19 cases was higher in the year 2021 compared to the previous year. This higher prevalence in 2021 compared to the year 2020 was probably due to the year-long movement control order in 2020 compared to a shorter duration of movement control order in 2021. For example, between March 18, 2020 and May 3, 2020, the Malaysian population underwent a full lockdown. People were strictly ordered to stay home, and schools, businesses, and offices were closed. Only essential services were allowed during this period. Following this period, the Conditional Movement Control Order (CMCO) was implemented from May 4, 2020 to June 9, 2020 and then the Recovery Movement Control Order (RMCO) was implemented from June 10, 2020 to March 31, 2021, during which time movement of the people was gradually eased and everyday activities returned to almost normal. In contrast, during 2021, there was only one period of total lockdown, which was between June 1, 2021 and June 28, 2021, following which the country resorted to having the National Recovery Plan (NRP) from June 15, 2021 to December 31, 2021, when again social and business activities were almost as per usual. Therefore, in the year 2021, the contact rate between the population of Malaysia was higher compared to the year 2020, hence this could explain why the number of cases was higher in 2021.

The results of this study also noted that most of the COVID-19 cases were reported in the central region of Malaysia (Selangor, Kuala Lumpur, and Putrajaya) (8). This geographical preponderance could be because the central region is the most developed and populous area of the country. According to the Department of Statistics, Malaysia, Selangor are the most populous states, while the most densely populated states are Kuala Lumpur (7,188 persons per square kilometer) and WP Putrajaya (2,354 persons per square kilometer). Higher population density can result in an increased contact rate, hence explaining the high incidence of COVID-19 in Selangor, Kuala Lumpur, and Putrajaya. A recent local study also showed that the central region had the strongest correlation between the COVID-19 cases and population density (*r* = 0.912; 95% CI 0.911, 0.913; *p* < 0.001). The propagation effect and the spread of disease were greater in urbanized districts or cities (9). Understandably, due to the high prevalence of cases, the number of deaths due to COVID-19 was also highest in these three states (Selangor, Kuala Lumpur, and Putrajaya) (1, 2, 8).

The results of this study showed that the proportion of patients who died from COVID-19 was not vaccinated, concurring with other studies (11, 12). Indeed, vaccination plays one of the most significant roles in reducing the infection risk and the severity of COVID-19 in humans (10, 13, 14). Malaysia began a massive nationwide COVID-19 vaccination program on February 2, 2021. The vaccination program, however, was not compulsory. Citizens and people living in Malaysia were encouraged to get themselves vaccinated. Civil servants and healthcare workers were highly encouraged, as they were workers of the essential services group. People who had contraindications for the vaccine, such as being allergic to the vaccine or its components, were exempted from this campaign. Although COVID-19 vaccination was not compulsory, those who have been vaccinated were given several privileges such as the ability to do interstate traveling or attend congregational prayers at religious sites. The COVID-19 vaccines in Malaysia were administered free of charge at numerous vaccination centers nationwide. In remote areas of the country or among people with disabilities and poor access, mobile vaccination teams were deployed to deliver the vaccines at home. Vaccination was also given at workplaces such as factories. The program was a success because by October 10, 2021, just 8 months after it began, approximately 90% of the adult population had received at least one dose of the COVID-19 vaccine. Consequently, the number of COVID-19 deaths decreased dramatically. Nonetheless, despite the good coverage of the vaccination program, there were still individuals who were not vaccinated against COVID-19 and these individuals make up the majority of the BID cases in Malaysia.

The data also showed that the majority of patients with COVID-19 in Malaysia had comorbidities and were among the elderly, both the findings concur with other global studies (8, 11, 15, 16). Many studies have demonstrated that people with diabetes, hypertension, obesity, and the elderly are vulnerable to COVID-19 (8, 11, 15, 16). As Malaysia is one of the countries with a high prevalence of non-communicable diseases [the prevalence of diabetes in the population was 36.6%, hypertension (30.0%), high cholesterol (38.0%), one in four people were physically not active, and one in two people was overweight or obese (17)], it could explain why those who had comorbidities make up a large proportion of those who died from COVID-19 in Malaysia during this study period.

### Brought-in-Dead Cases in Malaysia

Of the mortality cases during this study period, one in five (20%) were BID cases, the majority had no history of COVID-19 vaccination, non-Malaysian, were aged 18–59 years, and had no comorbidities.

The high prevalence of BID may be due to several factors, one of which could be the home quarantine measures. In Malaysia, at the beginning of the COVID-19 pandemic, all patients with COVID-19 were hospitalized. However, as the number of cases increased and the resources were limited, home quarantine for stable cases was implemented. The Malaysian government used the following classification of the COVID-19 clinical stages: (1) asymptomatic; (2) symptomatic, no pneumonia; (3) symptomatic, pneumonia; (4) symptomatic, pneumonia, and requiring supplemental oxygen; and (5) critically ill with multiorgan involvement. Individuals diagnosed with COVID-19 categories 1 and 2 were allowed to be quarantined out-of-hospital, either at their own homes, or, if the home situation is not suitable, the individual can be lodged at mass quarantine centers. Those who were hospitalized comprised patients in categories 3–5. The out-of-hospital quarantine measures began in early 2021, when the COVID-19 Assessment Centers (CACs) were setup in every district to assess which COVID-19 cases can undergo isolation at home. As of January 22, Malaysia had established 213 CACs nationwide (18). COVID-19 home isolation criteria include: the case is not displaying any COVID-19 symptoms, the case does not belong to the high-risk group (e.g., senior citizens, patients with chronic illness), the case has a suitable caretaker, and the residence is suitable for self-isolation. In addition, there were basic guidelines for patients placed under home isolation: the patient must stay alone in separate rooms, preferably with an attached toilet. If toilets must be shared, all the surfaces touched by the patient must be cleaned thoroughly, windows should be opened to ensure proper ventilation, and cases should not receive any visitors. In addition, face-to-face interactions with others in the household must be avoided and the person placed under isolation must report their status daily through the nation's COVID-19 mobile phone application called MySejahtera or to the medical officer in charge.

After the nationwide home quarantine measures, several factors could have led to the high prevalence of BID. For example, ignorance of the patient and/or the family members/caregivers of the COVID-19 disease progression could be a contributing factor. This ignorance may have made them unaware of the danger signs of COVID-19 and, consequently, succumbed to the infection at home, instead of going to the hospital. Another possibility was that the patient may not have known how to get to the hospital when the symptoms got worse, for example, not knowing that he can call for an ambulance for help. Another possible factor was that young patients did not know that they suffered from a chronic disease such as diabetes or hypertension and, hence, when they were infected with the COVID-19 virus, the patient deteriorated very quickly and did not get to the hospital in time. Inappropriate or delayed healthcare-seeking behavior has been shown to reduce the chances of getting immediate treatment, resulting in unfavorable outcomes (19).

The results of this study also showed that the majority of BID cases in Malaysia during this study period were among those who were relatively young (18–59 years old) similar to another nationwide study in the USA, which found that the percentage of out-of-hospital deaths among the elderly was low (only 33%) (18). This phenomenon could be because elderly patients were mostly hospitalized. Nonetheless, it is unclear exactly why a large proportion of BID victims in Malaysia are younger.

The data showed that non-Malaysians were more likely to be BID compared to Malaysians. Non-Malaysians can be categorized into legal vs. illegal workers/migrants. Legal workers, including students, are usually covered by medical insurance and are usually free to travel within the country without fear of getting arrested or detained. However, workers who are in the country illegally may not have any financial coverage for medical care and they are usually afraid of traveling albeit to a medical facility for fear of being arrested or detained. To address these issues, in January 2020, the Ministry of Health, Malaysia indicated that migrant workers who were suspected of positive COVID-19 or with close contact with patients with COVID-19 were exempted from paying the fees (i.e., registration, examination, treatment, and ward fees) at government facilities. In addition, in March 2020, the government assured that undocumented migrant workers who were seeking care and medical test at government health facilities will not be arrested and detained (20). However, in May 2020, hundreds of undocumented migrant workers and refugees were arrested in a massive raid operation conducted in Kuala Lumpur where they were rounded up and subsequently detained in immigration detention centers (21). These turn of events could have contributed to these migrant workers not coming forth to the hospital, if their condition deteriorated. In addition, access barriers to healthcare services could have contributed to a high probability of BID among non-Malaysians. A local study by Loganathan, Rui, Ng, and Pocock (2019) found that healthcare services in Malaysia are often inaccessible to migrant workers due to financial constraints, the need for legal documents such as valid passports and work permits, discrimination and xenophobia, physical inaccessibility, and employer-related barriers. In addition, language barriers may affect the quality of care received by migrant workers, by inadvertently resulting in medical errors, while preventing them from giving truly informed consent (22).

As far as the author is aware, this is the first study comparing the characteristics of inpatient deaths and BID cases using national data in Malaysia. Apart from highlighting the factors associated with the higher likelihood of BID, it is also important to emphasize that the percentage of BID cases in Malaysia during this period was higher than those reported in the USA and the UK (4%−8%) as mentioned earlier (1, 6, 7). The high percentage of BID cases is alarming and calls for urgent attention by the government, as it can signal the presence of gaps or weaknesses in the country's health system, either in the upstream state (access to vaccination) or downstream (access to medical care after being infected with COVID-19). Further studies should be conducted to explore in more depth the factors contributing to BID cases.

There are several limitations to this study. First, the comorbidity status of certain BID cases was not readily known, hence rendering the prevalence of comorbidity in the BID sample lower than that of the inpatient deaths. Second, it was not apparent in the database used in this study, whether the positive diagnosis of COVID-19 among the cases was done before or after postmortem examination. Hence, more detailed investigations on the characteristics of the BID and inpatient death cases should be carried out in the future to enhance an understanding of why BID occurred. This study is descriptive and could not show any causal relationship between the variables analyzed. Last but not least, the exogenous variable such as the policy of COVID-19 and hospital-related variables is suggested to include in the analysis, which might affect the BID occurred.

## Conclusion

One in five COVID-19 deaths in Malaysia between March and November 2021 was brought-in-dead (BID) cases. These cases were more likely to be among the younger age group, having no comorbidities, non-Malaysian, not vaccinated, and the interval between the date of death and diagnosis.

This high prevalence of BID is an alarming public health issue, which needs urgent attention by all the parties involved in the management of patients with COVID-19, namely, the patients themselves, the patients' families and caregivers, the community, healthcare workers, and even non-governmental organizations. Based on the findings of this study, there is a need to increase the intensity of the vaccination campaign, address any issues faced by non-citizens concerning COVID-19 management in- and out-of-hospital, increase the awareness of signs and symptoms of worsening COVID-19 among the population, emphasize the significance of self-monitoring among the patients especially those who undergo home quarantine, and determine the potential gaps in the health system that may contribute to their increased risk of deaths.

## Data Availability Statement

Publicly available datasets were analyzed in this study. This data can be found here: github.com/MoH-Malaysia/covid19-public; https://github.com/CITF-Malaysia/citf-public/tree/main/vaccination.

## Author Contributions

Conceptualization and writing—reviewing and editing: PYL, SAM, SMS, HKS, AZFA, and AM. Methods: PYL, SAM, SMS, HKS, and AZFA. Formal analysis: PYL, SAM, and AM. Writing—original draft preparation: PL and AM. All the authors have read and agreed to the published version of the manuscript.

## Conflict of Interest

The authors declare that the research was conducted in the absence of any commercial or financial relationships that could be construed as a potential conflict of interest.

## Publisher's Note

All claims expressed in this article are solely those of the authors and do not necessarily represent those of their affiliated organizations, or those of the publisher, the editors and the reviewers. Any product that may be evaluated in this article, or claim that may be made by its manufacturer, is not guaranteed or endorsed by the publisher.
